# Introduce a novel post-biotic against* Pseudomonas aeruginosa* biofilm formation using* Escherchia coli Nissle1917* outer membrane vesicles

**DOI:** 10.1186/s13104-023-06504-x

**Published:** 2023-09-09

**Authors:** Maryam Alaei, Fatemeh Aghakhani, Sarvenaz Falsafi, Hoora Mazaheri, Ava Behrouzi

**Affiliations:** 1 Department of Microbiology, Faculty of Advanced Science and Technology, Tehran Medical Science, Islamic Azad University, Tehran, Iran; 2 Department of Molecular Biology, Pasteur Institute of Iran, Tehran, Iran

**Keywords:** *algD*, *EcN*, *PpyR*, *Pseudomonas aeruginosa*, OMVs

## Abstract

*Pseudomonas aeruginosa* is an opportunistic bacterial pathogen that can cause acute infections as well as chronic ones in humans. The expression of *algD* and *PpyR* genes involved in biofilm formation in clinical isolates of *P. aeruginosa* in the presence of *Escherichia coli Nissle1917* outer membranes vesicles (*EcN* OMVs) was evaluated. All isolates were tested for biofilm formation. qPCR and disk diffusion were used to identify the expression of *algD* and* PpyR* genes, and antimicrobial resistance, respectively. *EcN* OMVs caused a more significant loss of* algD* and* PpyR* expression, compared with the control group. *EcN* OMVs contain a variety of biomolecules that are capable of influencing the biofilm formation genes. *EcN* OMVs treatment reduced* P. aeruginosa* biofilm formation significantly, which emphasizes their positive role in inhibiting biofilm formation. As a result, *EcN* OMVs can be used as new therapeutic strategies for inhibiting *P. aeruginosa* biofilm formation.

## Introduction

As a Gram-negative bacterium,* Pseudomonas aeruginosa (P. aeruginosa)* causes nosocomial infections as well as fatal infections in cancer patients, those who have undergone surgery, those with severe burns, and those with human immunodeficiency virus* (HIV)* [1, 2]*.* The adaptability and intrinsic antibiotic resistance of *P. aeruginosa* often limit the efficacy of common antimicrobial agents like antibiotics and increase the mortality rate. Furthermore, due to the increasing incidence of multidrug-resistant (MDR) strains, medical therapy against this pathogen has become complicated [3, 4]. *P. aeruginosa’s* ability to form biofilms plays a major role in chronic infections [5]. *P. aeruginosa* uses biofilm formation to survive harsh environments such as antibiotic exposure and host immunity. According to the National Heart, Lung, and Blood Institute, up to 80% of all bacterial infections are caused by biofilms. At least three different exopolysaccharides make up the biofilm components of *P. aeruginosa*, including* alg D*, *Psl*, and *Pel* [6]. By increasing exopolysaccharides from the *psl* operon and enhancing pyoverdine synthesis, Pseudomonas quinolone signal; 2-heptyl-3-hydroxy-4(1H)-quinolone (PQS) production, and elastase activity, the PA2663 (PpyR) gene product increases biofilm formation and reduces swarming and swimming motility. In addition to living cells, probiotics are also believed to have beneficial effects through bacterial metabolic byproducts. Post-biotics are soluble factors generated by live bacteria or molecules released into the environment that provide health benefits directly or indirectly [7]. Outer membrane vesicles (OMVs) released by pathogens have been studied extensively, and their role in virulence has been proven. However, vesicles released by commensal and probiotic bacteria ( post-biotics) have been found to have beneficial effects on the host [8, 9]. OMVs are considered as a kind of vehicle for cell-to-cell signaling in various bacteria and play an important role in survival and adaptation. Post-biotics—also known as meta-biotics, biogenics, or simply metabolites—are soluble factors, secreted by live bacteria, or released after bacterial lysis providing physiological benefits to the host. *P. aeruginosa* infections are known to be controlled by reducing biofilm formation. The purpose of this study was to determine if OMV, a post-biotic obtained from *E coli Nissle1917*, could interfere with the mechanisms of biofilm formation by *P. aeruginosa*. OMVs were found to have potent anti-biofilm properties when applied to pre-formed biofilms of *P. aeruginosa*. It seems that OMVs could be an effective means to combat biofilm-forming microorganisms.

## Materials and methods

### Preparation of *E. coli Nissle1917*

*E. coli Nissle1917* was obtained from Mutaflor tablets (Pharma-Zentrale GmbH, Herdecke, Germany). Luria–Bertani (LB) broth was used to cultivate the bacteria for 24 h (h) at 37 °C. The bacteria were then inoculated into brain heart infusion broth (Quelab, Canada) and gently shaken at150 rpm for 10–12 h until the optical density (OD600) reached 1. Bacterial pellets were centrifuged (11,000 g for 20 min) and washed twice with phosphate-A buffered saline (PBS).

### OMVs isolation

The supernatant was filtered, and OMVs were extracted by ultracentrifugation at 200,000 g for 2 h at 4 °C, as previously described [10]. Re-suspension of the pellets in PBS was performed and they were stored at – 80 °C. Using scanning electron microscopy (SEM), the morphology of the OMVs was determined, and then the protein sample pattern was analyzed using sodium dodecyl-sulfate polyacrylamide gel electrophoresis (SDS-PAGE). Following the manufacturer’s instructions, lipopolysaccharides (LPS) were measured in OMVs using the Limulus amebocyte lysate (LAL) Chromogenic Endotoxin Quantitation Kit(Thermo Fisher Scientific, United States).

### Culture conditions for *Pseudomonas aeruginosa*

Various clinical samples were provided from the microbial bank of medical branch of Islamic Azad University between 1 September 2021 and 28 February 2022 after receiving ethical approval from the Institutional review committee. Clinical *P. aeruginosa* cultures were grown overnight at 37 °C on tryptic soy agar (TSA) (Merck Millipore- cat num: 105458) plates from − 80 °C glycerol stocks. A single colony was then inoculated into 10 ml of tryptic soy broth (TSB) and incubated overnight at 37 °C. Microbiological and biochemical methods were used for the identification of *P. aeruginosa* isolates in the laboratory, including pigment production in agar, oxidase and catalase tests, reactions in triple sugar iron (TSI) agar, SIM (sulfide indole motility) and oxidative-fermentative (OF) media (Merck, Darmstadt, Germany), followed by growth at 42 °C. Following the Clinical and Laboratory Standards Institute (CLSI) recommendations, the susceptibility of isolates to different antibiotics was determined by disk diffusion agar on cation-adjusted Mueller–Hinton agar (Merck, Darmstadt, Germany). Ceftazidime (CAZ, 30 μg), piperacillin/tazobactam (PTZ, 100 μg/10 μg), ciprofloxacin (CIP, 5 μg), levofloxacin (LEV, 5 μg), gentamicin (GM, 10 μg), amikacin (AK, 30 μg), tobramycin (TOB, 10 μg), imipenem (IMI, 10 μg), and meropenem (MEM, 10 μg) were all effective antibiotic disks (MAST Diagnostics, Merseyside, UK). As a control*, P. aeruginosa* ATCC 27853 was used for susceptibility testing*.* Multidrug-resistant strains were chosen since antibiotic resistance and biofilm formation is directly related [11]. The term multidrug-resistant *P. aeruginosa* (MDR-PA) refers to the isolates resistant to more than one antimicrobial agent in three or more categories [12].

### Biofilm formation

The colorimetric microtiter plate assay was used to assess biofilm formation quantitatively. 12 out of 50 highly resistant strains were selected for biofilm formation. Results confirmed that antibiotic resistance is associated with biofilm formation [13, 14], therefore, MDR-PA was selected for biofilm formation*. P. aeruginosa* culture was adjusted to McFarland standard turbidity of 1. After dilutions of 1:100 in 200 µl TSB containing 1% glucose (Merck, Darmstadt, Germany), suspensions were transferred into sterile polystyrene microplates with OMVs (50 µg) or without OMVs. After 24 h of incubation at 37 °C, wells were gently washed three times with sterile PBS at pH 7.3. Adherent biofilms were fixed in 99% methanol for 15 min, and then the solutions were removed, and the plate was air-dried. 200 µl of crystal violet 0.1% (Sigma Chemical Co., St Louis, MO, USA) was used to stain biofilms for 5 min at room temperature, followed by rinsing and drying the biofilms. Treatment with 200 µl of 95% ethanol for 30 min destained the biofilm in each well. Microtiter plate readers (BioTek, Bad Friedrichshall, Germany) were used to measure OD. *P. aeruginosa* ATCC 27853 with OMVs treatment was utilized as a positive control and *P. aeruginosa* ATCC 27853 without OMVs was utilized as a negative control. The experiments were conducted in triplicate [5].

### Real-time PCR

RNA extraction was performed on both treated and control samples. The manufacturer's instructions were followed to synthesize cDNA after TRIzol reagent quality was confirmed after 24 h. Real-time PCR was performed using SYBRGreen method, and 16srRNA was used as a reference gene. Table 1 lists the primer sequences. In PCR, SYBR green is added to create a fluorescent signal by binding to the double-stranded DNA. Initial denaturation was performed at 95 °C for 10 min and then it applied at 95 °C, 57 °C, and 72 °C for 30 s for 35 cycles. The final extension was applied at 72 °C for 10 min.

**Table 1:** Sequence of primers used in qPCR

Forward PpyR [29]	Reverse PpyR
5-CGTGATCGCCGCCTATTTCC -3	5-ACAGCAGACCTCCCAACCG -3
Forward algD [30]	Reverse algD
5-GCGACCTGGACCTGGGCT-3	5- TCCTCGATCAGCGGGATC-3
Forward16srRNA08:00 AM	Revers16srRNA
5′-GAGGAAGTTGGGGATGACGT-3′	AGGCCCGGGAACGTATTCAC-3′ 5

### Statistical analysis

For statistical analysis, GraphPad Prism 8.0 (GraphPad Software Inc., CA, United States) was used. A two-tailed Student’s t-test or one-way ANOVA with Bonferroni’s post-hoc test was used. Statistical significance was defined as a *P value* less than 0.05.

## Results

### Antibiogram

**Fig. 1:**
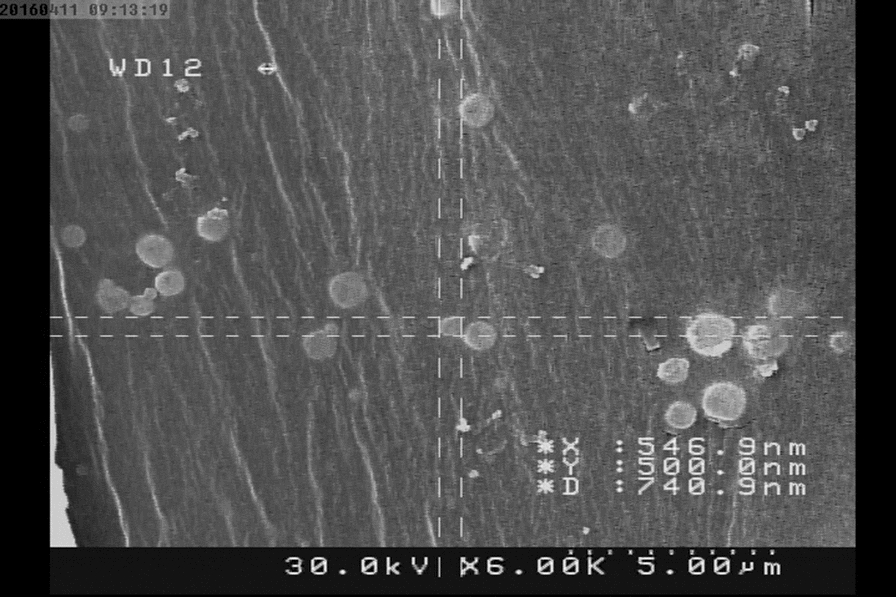
SEM micrographs of *EcN* OMVs

According to CLSI interpretive criteria [15], the resistance rate of *P. aeruginosa* isolates to tested antibiotics (Fig. 1) was as follows: IMI 100% (n = 15), MEM 80% (n = 12), GM 100% (n = 15), TN 80% (n = 12), AK 100% (n = 15), CIP 100% (n = 15), LEV 100% (n = 15), CAZ 100% (n = 15), and PTZ 100% (n = 15). The prevalence of MDR-PA and non-MDR-PA was 100% (n = 15) and 100% (n = 15), respectively. MDR-PA was selected for further studies.

### Characteristics of OMVs by FE-SEM

SEM analysis indicated that the majority of *EcN* OMVs consisted of spherical shape with a diameter of 40 to 150 nm (Fig. 1). The structural integrity of OMVs remained intact during extraction.

### Minimum inhibitory concentration (MIC)* P. aeruginosa* biofilm formation

**Fig. 2:**
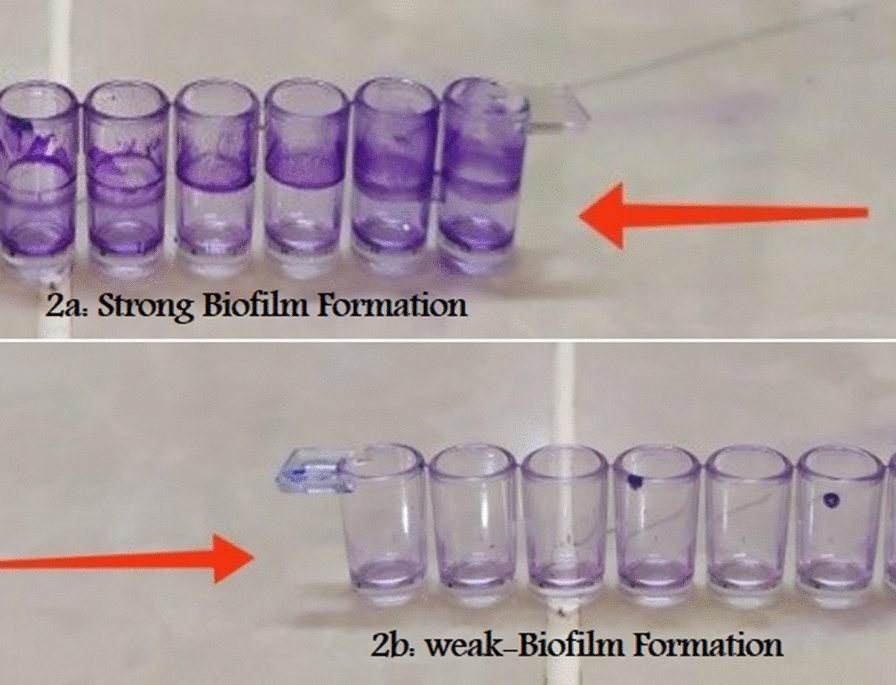
96-well tissue culture plate showed, strong biofilm producers differentiated by crystal violet stain. **b** 96-well tissue culture plate showed weak-biofilm producers in the presence of *EcN* OMVs differentiated by crystal violet staining

**Fig. 3:**
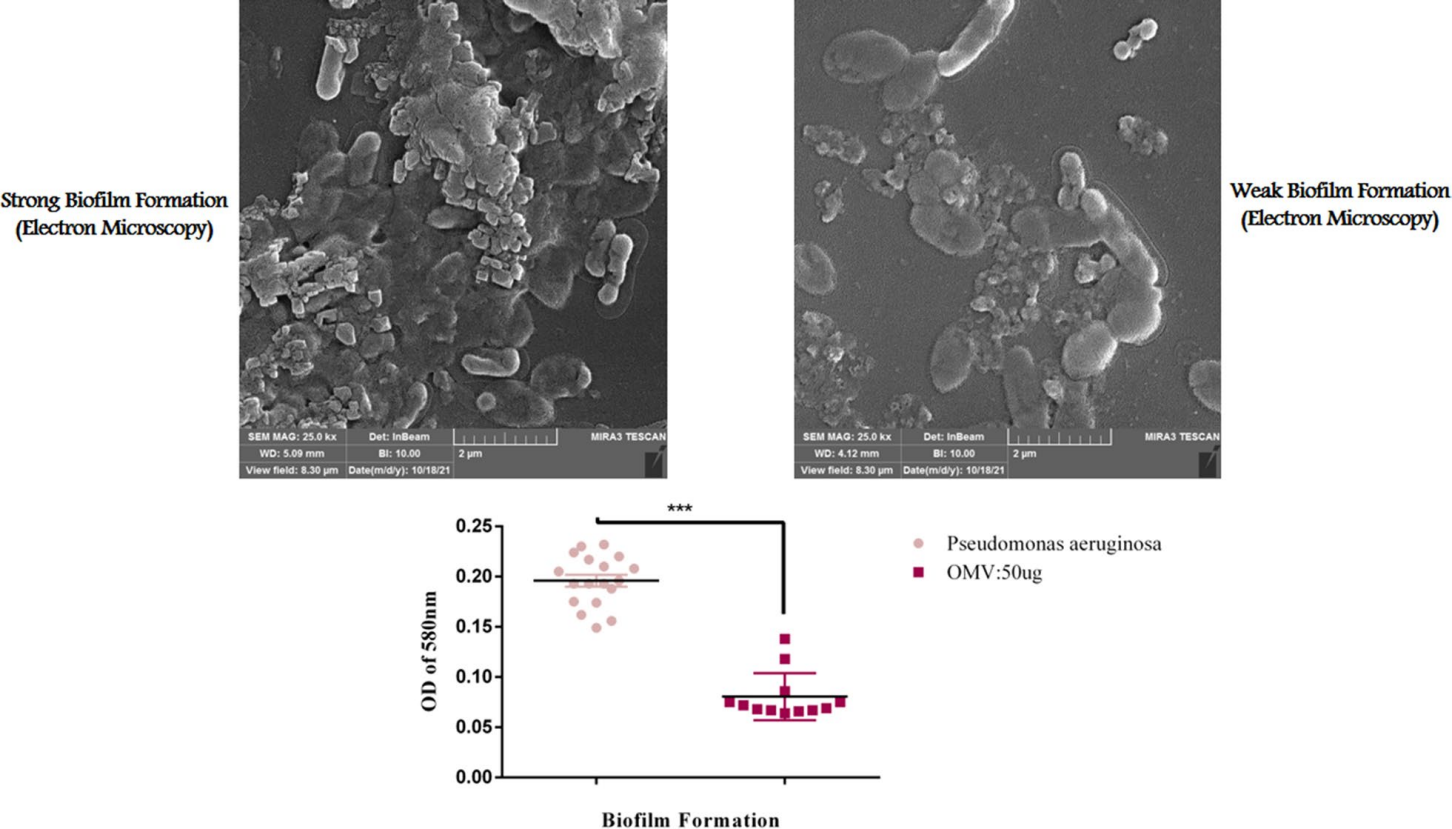
Mean + / − SEM of biofilm mass analysed by CV staining (OD_570_). *Pseudomonas aeruginosa* cultured for 24 h in the presence and absence of *EcN* OMVs were assessed. *EcN* OMVs were tested in the presence and absence of* Pseudomonas aeruginosa* cultures for 24 h*.* Values of *p* < 0.05 (*), *p* < 0.001 (***) and *p* < 0.0001 (****) were considered significant

The MIC for OMVs was 50 µg. Biofilm formation was not observed in the control group at day 0 and the day before incubation. The control group was only treated with PBS. Two different concentrations of OMVs were selected for this study; high-dose (50 µg), and medium-dose (25 µg). The medium-dose group formed the most biofilms, compared with the high-dose group. The biofilm formed on the glass tube of the medium-dose group was visible from the high-dose group; in contrast, the biofilm formed on the glass tube of the control group was visible from both the medium and high-dose groups (Figs. 2 and 3). Biofilm formation and multidrug resistance rate were positively correlated. A direct correlation exists between OMV treatment and non-OMV treatment in terms of biofilm formation.

### Real-time PCR (evaluation of *algD* and *PpyR* expression)

**Fig. 4:**
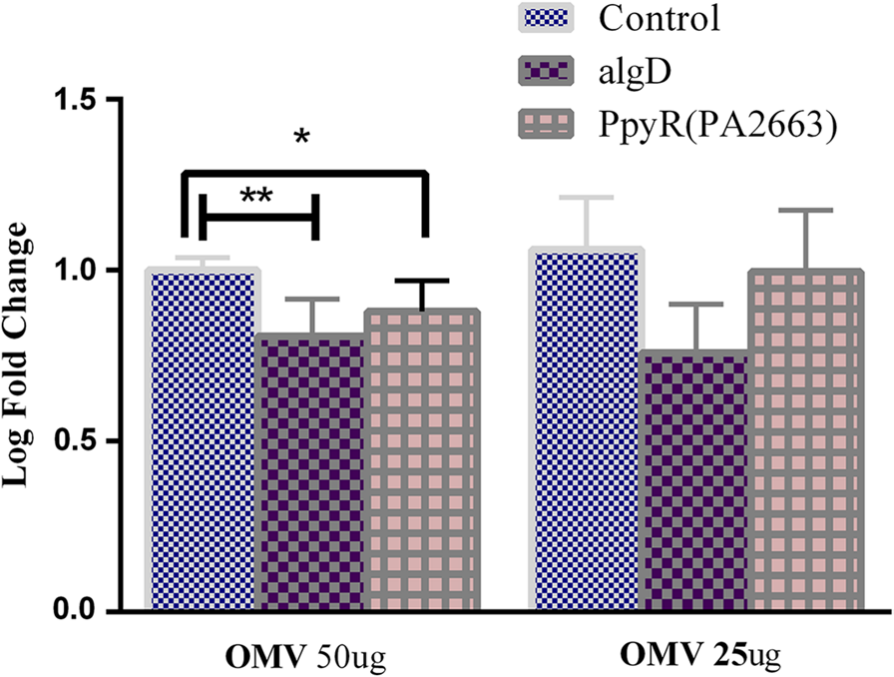
Real-time analysis of *P. aeruginosa* biofilm formation in the presence and absence of *EcN* OMVs. *'P < 0.05 and P < 0.01 were considered statistically significant, respectively. As an internal control, 16srRNA was used*. EcN* OMV effects on *algD* and *PpyR* genes. A 25 µg and 50 µg concentration of *EcN* was used to treat *pseudomonas aeruginosa* in a clinical study

Antibiotic resistance is widely known to be directly related to the formation of biofilms. Therefore, we can control the formation of biofilms and inhibit a variety of infections by determining the effect of novel antibacterial components. After obtaining the best concentration of OMVs by diluting different concentrations by MIC, OMVs were treated with biofilm-forming *P. aeruginosa* and then the gene expression was measured by qPCR. A significant decrease in* algD* gene expression was observed in* P. aeruginosa* biofilms*.* OMVs at 50 µg/ml reduced *algD* expression, but those at 25 µg/ml did not (Fig. 4). The expression of *algD* was not affected by OMVs at the concentration of 25 µg/ml.

*PpyR* expression at the mRNA level decreased in response to* EcN* OMVs at a concentration of 50 µg/ml*.* The expression of *PpyR* was not affected by OMVs at the concentration of 25 µg/ml. It appears that *EcN* OMVs have a dose-independent effect on the studied genes (Fig. 4).

## Discussion

*P. aeruginosa* is regarded as an opportunistic human pathogen, which can attach, colonize, invade the local area, and spread as a systemic disease [15, 16]. The emergence of resistant microorganisms to a variety of antibiotics has become a major health concern worldwide. The majority of chronic *P. aeruginosa* infections are caused by the bacteria’s ability to form biofilms that confer resistance to antibiotics. For instance, Bhandari et al. found that all MDR *P. aeruginosa* isolates formed biofilms to varying degrees. Conventional antibiotic therapy cannot eradicate bacteria embedded in biofilms because they are more tolerant of antibiotics than their planktonic counterparts [17]. It was found that biofilm formation did not differ between MDR and non-MDR *P.aeruginosa* [18]. In order to control antimicrobial resistance, discovering an effective treatment against *P. aeruginosa*-associated infections is necessary. It is important, however, to identify more effective antibiofilm agents to treat *P. aeruginosa*-associated infections, since these infections are complex and difficult to be treated easily. Today, biofilm infection therapy is challenging for clinicians. Biofilm-related infections cannot be effectively treated with antibiotics; but understanding how biofilms work can assist us in fighting them [18, 19]. Thus, today many studies are looking for new approaches to overcoming biofilm formation. During the past few years, biofilm formation and pathogenesis have been extensively studied. Attila et al.showed that PA2663 (PpyR) increased biofilm formation in *Pseudomonas aeruginosa* PAO1 through the *psl* operon and stimulated virulence and quorum-sensing phenotype. They demonstrated that mutants in the *PpyR* gene in *P. aeruginosa* formed 20-fold and 11-fold fewer biofilms in Luria–Bertani (LB) and LB glu media after 24 h, respectively, and inactivated *PpyR* (PA2663) reduced *P. aeruginosa* virulence toward barley [18, 20]. In the current study, *EcN* OMVs reduced the expression of *PpyR* gene significantly (50 µg). The insufficient supply of antibiotics and the emergence of antibiotic resistance mechanisms suggest that introduction of a new sufficient component could open new doors for medicine. *algD* is another important gene involved in *P. aeruginosa* biofilm formation and antibiotic resistance. According to Rajabi et al., the *algD* gene is present in 78.6% of* P. aeruginosa* strains and it plays an important role in biofilm formation [19]. Our data showed that the expression of *algD* expression reduced significantly in the presence of *EcN* OMVs (50 µg). A phenotypic evaluation of biofilm formation found that *EcN* OMVs reduced the biofilm formation in comparison with controls (samples without *EcN* OMVs). In the current study, the reduction of *algD* and *PpyR* expression in the presence of OMVs correlated significantly with decreased biofilm formation. In light of these results, it is possible to introduce novel components that inhibit the growth of biofilms in *P. aeruginosa*. Further evaluation of *EcN* OMVs is needed to find their mechanism of inhibition. Post-biotics have antimicrobial, antioxidant, and immunomodulatory properties as well as beneficial physiological, immunological, neurohormonal, and regulatory effects [21]. Considering the inadequacy of current approaches, new biofilm-fighting strategies would be beneficial in the clinic. Research has indicated that probiotics and post-biotics can combat infectious biofilms. In addition to the secretion of antagonistic substances such as surfactants, bacteriocins, exopolysaccharides (EPS), organic acids, lactic acid, fats, enzymes (amylase, lipase) and hydrogen peroxide, these molecular mechanisms also result in the creation of unfavorable environmental conditions for pathogens including pH changes as well as competition for surfaces and nutrients [22]. It seems that OMVs with various components can effectively change the environmental condition and they create an improper situation for attached pathogenic bacteria and biofilm formation. In addition to being versatile containers, OMVs can also be loaded with nucleic acids and other components [23–25]. Wang et al*.* demonstrated that OMV treatment reduced the biomass, biofilm integrity, and viability of *S. mutans* biofilm cells in a dose-dependent manner [26]. The capacity of different *Lactobacillus* species to inhibit the growth, biofilm formation, and gene expression of *S. mutans* was assessed. Susceptibility testing confirmed the antibacterial (pH-dependent) and anti-biofilm activities of *L. casei* (ATCC 393), *L. reuteri* (ATCC 23272), *L. plantarum* (ATCC 14917), and *L. salivarius* (ATCC 11741) against *S. mutans* [27]. Post-biotics are substances released by microorganisms during their metabolic activity that have a beneficial effect on the host. The risks associated with the intake of post-biotics are minimized because they do not contain live microorganisms [28]. In view of this, OMVs have the potential to be further evaluated against pathogenic bacteria as a novel post-biotic. The results indicate that OMVs derived from *EcN* may be an effective way to change key gene expression to reduce the formation of biofilms in a dose-dependent manner. Thus, it appears that it may utilize as an alternative component for antibiotics.

## Conclusion

Infectious diseases can be caused by *Pseudomonas* biofilms, which are usually very readily formed by these bacteria. As a result, *P. aeruginosa* mucoid growth has caught the attention of researchers due to its association with biofilm formation. One of the major drawbacks of treating biofilm-related infections is that they are resistant to antibiotics that are already available. Accordingly, *EcN* OMVs appeared to be a promising new approach for treating *Pseudomonas* biofilms.

## Limitation

A better understanding of biofilm formation and genes involved might be gained through the analysis of other related genes in the presence of *EcN* OMVs.Additionally, fusing the first line antibiotic outside of OMVs and evaluating its effect on biofilm can be useful, since the OMVs can be used to better deliver the antibiotic inside.

## Data Availability

All data generated or analysed during this study are included in this published article and its Additional files.

## Abbreviations

CAZ: Ceftazidime
PTZ: Piperacillin/Tazobactam
CIP: Ciprofloxacin
LEV: Levofloxacin
GM: Gentamicin
AK: Amikacin
TOB: Tobramycin
IMI: Imipenem
MEM: Meropenem
EcN: E. coli Nissle 1917
MDR: Multi drug resistance
OMVs: Outer membrane vesicles
